# Risk of febrile neutropenia in breast cancer chemotherapy despite the prophylactic use of pegfilgrastim: a retrospective analysis

**DOI:** 10.1007/s12282-026-01907-7

**Published:** 2026-08-14

**Authors:** Hiroki Kusama, Yoshiya Horimoto, Kyoko Iwai, Yuki Kitaoku, Fujina Takeda, Ryo Hamaoka, Minami Iwai, Mizuki Mizuuchi, Tomoka Ushio, Kyoko Orimoto, Takashi Ishikawa

**Affiliations:** Department of Breast Surgical Oncology, Tokyo Medical University, 6-7-1 Nishishinjuku, Shinjuku-ku, Tokyo, 160-0023 Japan

**Keywords:** Breast cancer, Pegfilgrastim, Febrile neutropenia, Predictive factors

## Abstract

**Background:**

Pegfilgrastim, a long-acting granulocyte colony-stimulating factor (G-CSF), is used to prevent febrile neutropenia (FN) in breast cancer patients undergoing chemotherapy. However, a small subset of patients still develops FN. This study aimed to assess the real-world incidence of FN and identify potential risk factors in this specific population.

**Methods:**

This retrospective study included breast cancer patients who received pegfilgrastim prophylaxis at Tokyo Medical University Hospital between February 2019 and December 2024. Inclusion required pegfilgrastim use at least once during chemotherapy. FN was defined as an absolute neutrophil count below 500/µL (or a predicted ANC below 1,000/µL) and an axillary temperature 37.5 °C or higher. Cases of FN and associated risk factors were analyzed using logistic regression models.

**Results:**

This study included 378 patients (median age 53). Tumor subtypes were luminal (50.0%), HER2-positive (20.9%), and triple-negative (29.1%), with dose-dense regimens being the most common (55.8%). Of 2,784 total chemotherapy administrations, pegfilgrastim was used in 2,125 cycles (76.3%). The incidence of FN despite pegfilgrastim was 2.1% (8/378), with the highest rate observed in immune checkpoint inhibitor-containing regimens (9.1%, 2/22). Multivariate analysis identified a low baseline platelet count as a significant independent risk factor for combined FN and grade 3–4 neutropenia (OR: 0.98; 95% CI: 0.96–0.99; *P* = 0.02).

**Conclusion:**

Although pegfilgrastim is effective, FN still occurs in a small percentage of patients. A low baseline platelet count was associated with an increased risk of FN or severe neutropenia despite pegfilgrastim prophylaxis, though this finding requires confirmation in larger studies given the limited number of events.

**Supplementary Information:**

The online version contains supplementary material available at https://doi.org/10.1007/s12282-026-01907-7.

## Introduction

Chemotherapy remains a cornerstone in the treatment of breast cancer, and maintaining relative dose intensity (RDI) is crucial for maximizing therapeutic efficacy [1]. However, chemotherapy-induced febrile neutropenia (FN) not only increases the risk of sepsis, which can be life-threatening, but also leads to dose reductions or delays that can compromise patient outcomes [2]. FN primarily results from chemotherapy-induced myelosuppression, which suppresses bone marrow function and significantly reduces neutrophil counts, thereby elevating the risk of infection. Granulocyte colony-stimulating factors (G-CSF) promote the proliferation and differentiation of neutrophil precursors in the bone marrow, thereby accelerating neutrophil recovery and reducing the duration of neutropenia [3]. Initially, G-CSF were used as therapeutic agents to treat FN after its onset. Pegfilgrastim is a G-CSF that has been pegylated to extend its half-life, allowing it to suppress the occurrence of neutropenia with a single administration after chemotherapy. This advancement enabled G-CSF to be used prophylactically, thereby reducing the incidence of FN [4, 5].

In breast cancer, G-CSF are particularly recommended for high-risk regimens [6]. According to the National Comprehensive Cancer Network (NCCN) guidelines, risk factors include age over 65 years, prior chemotherapy or radiotherapy, neutropenia, bone marrow involvement by the tumor, poor performance status, and impaired renal or hepatic function [7]. Similarly, the American Society of Clinical Oncology (ASCO) guidelines emphasize additional factors such as advanced cancer, recent surgery, heart disease, and Human Immunodeficiency Virus (HIV) infection [8]. The European Organization for Research and Treatment of Cancer (EORTC) guidelines also list age over 65 years, prior FN, and lack of G-CSF use [9].

Despite the effectiveness of pegfilgrastim in preventing FN, a small number of patients still require hospitalization due to FN [4]. Few reports have examined the risk factors. The limited available evidence suggests that a low platelet count and administration of pegfilgrastim on the same day as chemotherapy is associated with an increased risk of febrile neutropenia [10, 11]. In addition, no studies specifically targeting breast cancer have been identified. This study specifically focuses on breast cancer patients who developed FN despite receiving pegfilgrastim. Understanding the potential for FN to occur despite the use of pegfilgrastim, as well as its risk factors, is crucial. It enables improved patient management by identifying high-risk individuals, allowing for closer monitoring and early intervention, thereby preventing deaths caused by FN. Additionally, it facilitates the optimization of treatment plans, such as chemotherapy schedules or implementing preventive medications tailored to individual patients, resulting in improved patient safety and outcomes.

## Methods

### Patients

This retrospective study included 378 breast cancer patients treated at Tokyo Medical University Hospital between February 2019 and December 2024. The inclusion criteria required that patients received pegfilgrastim at least once during chemotherapy administered with curative intent. This population consisted of patients with early breast cancer or those receiving treatment for locoregional recurrence. Patients treated solely for distant metastatic disease were excluded from this analysis. In addition, weekly chemotherapy regimens were excluded from the cycle-level analysis because pegfilgrastim is not indicated for weekly schedules.

### Data collection

Clinical data including pre-chemotherapy peripheral blood data were collected from electronic medical records. The cutoff for other blood data were set based on the institutional upper normal limit. Staging was based on clinical stages for patients receiving neoadjuvant chemotherapy (NAC) and on pathological stages for those who did not. Fever during chemotherapy was assessed based on the pre-chemotherapy questionnaire. Blood test data obtained before the initiation of the first chemotherapy cycle were used, representing the baseline data for each patient. In the case of local recurrent treatment, the baseline was defined as the first blood test results obtained before the initiation of chemotherapy with pegfilgrastim. If baseline blood test data were unclear due to factors such as hospital transfers, risk factor analysis based on blood test data was not conducted.

### Pathologic features

Estrogen receptor (ER) status were evaluated by immunostaining; TS (Allred score) < 3 was defined as a negative. Human Epidermal Growth Factor Receptor 2 (HER2) was evaluated according to the American Society of Clinical Oncology–College of American Pathologists guideline [12]. Immunostaining scores 0 and 1 + were considered negative, and 2 + was considered negative if no gene amplification was detected by in situ hybridization [13]. We defined ER-positive/HER2-negative cases as luminal, any ER/HER2-positive cases as HER2-positive, and ER-negative/HER2-negative cases as triple-negative breast cancer (TNBC).

### Classification of chemotherapy regimens

Administration of anthracycline and/or taxane agents on a biweekly schedule was classified as ‘dose-dense’. When administered every three weeks, it is referred to as ‘tri-weekly’. For regimens combining HER2-targeted therapy with chemotherapy, the designation ‘HER2-targeted therapy’ is used. Similarly, when immune checkpoint inhibitors (ICI) are combined with chemotherapy, the term ‘ICI’ is applied. Regimens that do not contain anthracyclines or taxanes were not included in this study.

### Pegfilgrastim administration and prophylactic approaches

Pegfilgrastim (3.6 mg) is administered subcutaneously at least 24 h after chemotherapy. Primary prophylaxis refers to the preventive use of pegfilgrastim from the first cycle of chemotherapy in cases of dose-dense regimens or high FN risk. Secondary prophylaxis is defined as the initiation of pegfilgrastim from the second chemotherapy cycle onward following an episode of FN or in response to an increased FN risk associated with chemotherapy-induced neutropenia. Because secondary prophylaxis is an option for some patients, some chemotherapy cycles are administered without pegfilgrastim.

### Definitions of FN

FN was defined as an absolute neutrophil count (ANC) below 500/µl, a predicted ANC below 1,000/µl, and an axillary temperature of 37.5 °C or higher [14]. Post-pegfilgrastim administration visits were not mandatory, and neutrophil counts were only taken when patients visited a hospital and had blood tests for any reason.

### Statistical analysis

Statistical analyses were performed using SPSS 31 software (IBM Corp., Armonk, NY, USA). Logistic regression models were used to identify factors associated with febrile neutropenia (FN). For the full-model analysis, age, ANC and platelet count were selected based on the sample size and the clinical relevance of the variables examined. The Wald test was used for univariate and multivariate significance tests. In all statistical analyses, *P* < 0.05 was considered significant.

## Results

### Patient characteristics

The median age of 378 patients was 53 years (range: 23–82) (Table 1). Of the 378 patients, 349 (92.3%) received neoadjuvant or adjuvant therapy for Stage I–III disease, while 29 (7.7%) were treated for locoregional recurrence. Tumor subtypes were categorized as luminal in 189 cases (50.0%), HER2 positive in 79 cases (20.9%), and TNBC in 110 cases (29.1%). Few cases had comorbid organ dysfunction, with diabetes observed in 16 cases (4.2%). NAC was administered to 240 (63.5%), adjuvant chemotherapy to 109 (28.8%), and treatment for locoregional recurrence to 29 (7.7%). The most common regimen was dose-dense, used in 211 patients (55.8%), followed by chemotherapy with HER2-targeted therapy in 77 (20.4%), tri-weekly in 66 (17.5%), chemotherapy with immune checkpoint inhibitor in 22 (5.8%), and others in 2 patients (0.5%). Pegfilgrastim was administered in all cases, with primary prophylaxis administered to 272 patients (72.0%), and secondary prophylaxis to 106 patients (28.0%). Because some patients received secondary prophylaxis, 659 chemotherapy administrations were given without pegfilgrastim.

**Table 1:** Baseline patient characteristics and laboratory data

Variables	*N* = 378 Cases (%)
Median age, years (range)	53 (23–82)
Age	
< 65 years	309 (81.7)
≥ 65 years	69 (18.3)
Stage*	
Ⅰ	67 (17.7)
Ⅱ	192 (50.8)
Ⅲ	90 (23.8)
Locoregional recurrence	29 (7.7)
Subtype	
Luminal	189 (50.0)
HER2 positive	79 (20.9)
TNBC	110 (29.1)
Baseline laboratory data**, median (range)	
White blood cell count (/µL)	5700 (2500–10800)
Neutrophil count (/µL)	3421 (1014–9110)
Lymphocyte count (/µL)	1618 (483–4380)
Hemoglobin (g/dL)	13.2 (6.8–16.9)
Platelet (×10⁴/µL)	252.0 (61.0–635.0)
AST (U/L)	
< 30	339 (89.7)
≥ 30	39 (10.3)
ALT (U/L)	
< 30	340 (89.9)
≥ 30	38 (10.1)
Cardiovascular disease	
No	372 (98.4)
Yes	6 (1.6)
Renal disease	
No	374 (98.9)
Yes	4 (1.1)
Liver disease	
No	374 (98.9)
Yes	4 (1.1)
Diabetes Mellitus	
No	362 (95.8)
Yes	16 (4.2)
Prior radiation therapy	
No	361 (95.5)
Yes	17 (4.5)
Purpose of chemotherapy	
Neo adjuvant	240 (63.5)
Adjuvant	109 (28.8)
Locoregional recurrence	29 (7.7)
Regimen	
Dose dense	211 (55.8)
Anthracycline and Taxane	198
Anthracycline only	13
Tri weekly	66 (17.5)
Anthracycline and Taxane	43
Anthracycline only	7
Taxane only	16
Chemotherapy with HER2-targeted therapy	77 (20.4)
Chemotherapy with Immune checkpoint inhibitor	22 (5.8)
Others	2 (0.5)
Purpose of administration	
Primary prophylaxis	272 (72.0)
Secondary prophylaxis	106 (28.0)

*Staging was based on clinical stage for patients receiving neoadjuvant chemotherapy and on pathological stage for those who did not

**Laboratory values represent baseline (pre-chemotherapy) measurements

HER2, human epidermal growth factor receptor 2; TNBC, triple negative breast cancer; AST, Aspartate Aminotransferase; ALT, Alanine Aminotransferase

### Incidence of febrile neutropenia and related outcomes

The total number of chemotherapy administrations was 2,784. Of these, pegfilgrastim was administered a total of 2,125 times (76.3%). Of the 2,125 pegfilgrastim administrations, FN occurred 8 times (0.4%). In contrast, among the 659 chemotherapy administrations without pegfilgrastim, FN occurred 21 times (3.2%); the incidence of FN was significantly higher in the non-pegfilgrastim group compared with the pegfilgrastim group (*p* < 0.01).

On a per-patient basis, FN were observed in 29 of 378 cases (7.7%), of which 21 cases occurred without pegfilgrastim. The incidence of FN with pegfilgrastim was 8 cases (2.1%) (Table 2). By subtype, the incidence was 3/189 (1.6%) in luminal cases, 2/79 (2.5%) for HER2 positive cases, and 3/110 (2.7%) for TNBC (Table 2). Analyzing by regimen, FN occurred in 3/211 (1.4%) in dose-dense regimens, 1/66 (1.5%) in tri-weekly regimens, 2/77 (2.6%) in anti-HER2 administration, and 2/22 (9.1%) in ICI, showing a higher incidence in the ICI regimen (Table 2). FN incidence appeared higher in the ICI group but statistical comparison was not performed due to the small number of events.

**Table 2:** Incidence of FN

	*n*/*N* (%)
Total FN	29/378 (7.7)
FN with pegfilgrastim	8/378 (2.1)
FN without pegfilgrastim	21/378 (5.6)
FN with pegfilgrastim by subtype	
Luminal	3/189 (1.6)
HER2 positive	2/79 (2.5)
TNBC	3/110 (2.7)
FN with pegfilgrastim by regimen	
Dose dense	3/211 (1.4)
Tri weekly	1/66 (1.5)
Chemotherapy with HER2-targeted therapy	2/77 (2.6)
Chemotherapy with Immune checkpoint inhibitor	2/22 (9.1)

FN, febrile neutropenia; TNBC, triple-negative breast cancer

When stratified by prophylaxis type, baseline characteristics differed significantly between groups in terms of subtype, ALT, and chemotherapy regimen (Supplementary Table 1). However, neither FN incidence (primary, 1.5%; secondary, 3.8%; *p* = 0.23) nor grade 3–4 neutropenia (3.3% vs. 4.7%; *p* = 0.55) differed significantly between the groups.

### Risk factors for febrile neutropenia and severe neutropenia

Next, we attempted to identify risk factors for FN with pegfilgrastim (Table 3). However, since there were only 8 FN cases, risk factor analysis could not be conducted. Therefore, risk factor analysis was conducted using FN + grade 3–4 neutropenia. Univariate analysis identified age (per 1-year; odds ratio [OR]: 1.05, 95% CI: 1.00–1.10, *p* = 0.04) and baseline platelet count (per 1 × 10⁴/µL; OR: 0.98, 95% CI: 0.97–0.99, *p* = 0.01) as significant factors. Multivariate analysis further demonstrated that baseline platelet count was an independent risk factor (OR: 0.98, 95% CI: 0.96–0.99, *p* = 0.02). Specifically, older age was significantly associated with risk in the univariate analysis only, whereas a low baseline platelet count remained an independent risk factor in the multivariate analysis.

Because the significance of perioperative chemotherapy for locoregional recurrence is unclear, we performed a sensitivity analysis excluding locoregional recurrence cases. The number of FN + severe neutropenia events decreased by one; however, platelet count remained an independent risk factor (Supplementary Table 2).

## Discussion

This study investigated the incidence of FN among breast cancer patients who received pegfilgrastim during chemotherapy. Despite the known efficacy of G-CSF in preventing FN, our findings revealed that 2.1% of patients developed FN. The occurrence of FN is extremely rare, making it difficult to identify risk factors. However, the combined analysis of FN and grade 3–4 neutropenia revealed that a decreased baseline platelet count was identified as an independent factor.

Previous large-scale clinical trials have reported FN rates of 1–2% in patients receiving pegfilgrastim prophylaxis [4, 5], with approximately 1% of cases being severe enough to require hospitalization [4]. Our findings align with these reports, indicating that the FN risk observed in real-world clinical practice is consistent with that reported in controlled trial settings.

General FN risk factors have been outlined in various guidelines. According to the NCCN guidelines, risk factors include age over 65 years, prior chemotherapy or radiotherapy, neutropenia, bone marrow involvement by the tumor, poor performance status, and impaired renal or hepatic function [7] Similarly, the ASCO guidelines emphasize additional factors such as advanced cancer, recent surgery, heart disease, and HIV infection [8]. The EORTC guidelines also list age over 65 years, prior FN, and lack of G-CSF or antibiotic prophylaxis as key risks [9]. In our study, none of these factors were identified as risk factors, except for the lack of G-CSF. With regard to prior radiotherapy in particular, 17 patients had received radiotherapy, mainly for a previous contralateral breast cancer. None of these patients developed FN or severe neutropenia. Because no events occurred in this subgroup, radiotherapy could not be included in the regression model.

Notably, our findings revealed a high incidence of FN in the ICI-containing regimen (9.1%), despite the limited number of cases. One of these two cases occurred during the first cycle of Pembrolizumab plus chemotherapy (epirubicin and cyclophosphamide); it was detected incidentally and did not require hospitalization. The other case was complicated by immune-related secondary adrenocortical insufficiency. To date, few studies have focused on FN risk specifically within ICI-containing regimens for breast cancer, and this area warrants further clinical investigation.

Existing literature on risk factors for FN despite G-CSF prophylaxis is relatively limited. One study found that administering pegfilgrastim on the same day as chemotherapy increased the risk of FN compared to administration on subsequent days [10]. Another study involving breast cancer and diffuse large B-cell lymphoma patients reported a 5.4% FN rate across 722 chemotherapy cycles, identifying a platelet count below 150,000 as a significant risk factor for FN [11]. These results are consistent with our findings, suggesting that a decreased baseline platelet count may be a significant risk factor of FN with pegfilgrastim.

When pegfilgrastim is used, indicators reflecting bone marrow function, such as platelet count, may be more important than overall systemic condition. This aligns with a redefined model of human hematopoiesis. In adult bone marrow, hematopoietic stem cells (HSCs) adopt a two-tier hierarchy, where they differentiate directly into specialized cells rather than passing through multiple intermediate stages. Notably, while the myeloid (neutrophil) lineage can be maintained by self-sustaining populations of these specialized cells, the megakaryocyte (platelet) lineage is uniquely dependent on direct branching from the HSC pool. Consequently, whereas neutrophil counts may be buffered by these resilient cellular layers, a low platelet count serves as a more direct and sensitive mirror of a diminished HSC reservoir [15]. Therefore, baseline platelet levels may more accurately reflect the bone marrow’s fundamental capacity to regenerate neutrophils following chemotherapy-induced damage. Filgrastim is internalized via the granulocyte colony-stimulating factor receptor expressed on neutrophils and neutrophil precursors and is subsequently degraded intracellularly [16]. As a result, it is unaffected by renal function, liver function, or age [17], which aligns with the findings of our study.

Our study has several limitations. First, due to the single-center, retrospective nature of this study, the number of FN cases was small (*n* = 8), and crucial clinical variables such as performance status could not be reliably collected or incorporated into the analysis. Given the rarity of FN, collaborative multi-center studies may be necessary in the future. Second, blood tests were routinely performed only during scheduled outpatient visits, rather than on a continuous or daily basis; therefore, mild, self-limiting febrile episodes at home or asymptomatic grade 3–4 neutropenia occurring between visits may have been overlooked, which could have led to an underestimation of the overall incidence of neutropenia and FN.

## Conclusion

Despite the use of pegfilgrastim, FN occurred in a small number of cases. In this study, a low baseline platelet count was suggested to be independently associated with severe neutropenia or FN under pegfilgrastim prophylaxis, suggesting the need for careful monitoring and individualized management in patients with low platelet counts. However, given the limited number of FN events, this finding should be interpreted with caution. Larger prospective cohort or multicenter studies are needed to validate the association between patient background factors, including platelet count, and the risk of FN under pegfilgrastim prophylaxis.

**Table 3:** Univariate and multivariate analyses of the clinical factors in association with FN + severe neutropenia with pegfilgrastim

	Univariate			Multivariate		
	OR	95% CI	*P* value**	OR	95% CI	*P* value**
Age: continuous (per 1 year)	1.05	1.00–1.10	0.04	1.04	0.99–1.09	0.14
Stage*: 3- (ref) vs. 1–2	5.86	0.76–45.36	0.09			
Chemotherapy History: No(ref) vs. Yes	3.72	0.41–33.39	0.24			
Adjuvant: Adjuvant(ref) vs. NAC	0.84	0.28–2.57	0.76			
Regimen: Dose dense(ref)						
Tri-weekly	0.80	0.09–7.25	0.84			
Anti-HER2	4.46	1.20–15.9	0.03			
ICI	5.20	0.90–30.0	0.07			
ANC: continuous (per 1/µL)	1.00	1.00–1.00	0.07	1.00	1.00–1.00	0.98
ALC: continuous (per 1/µL)	1.00	1.00–1.00	0.48			
Hemoglobin: continuous (per 1 g/dL)	1.10	0.71–1.71	0.68			
Platelet: continuous (per 1 × 10⁴/µL)	0.98	0.97–0.99	0.01	0.98	0.96–0.99	0.02
AST: < 30 vs. ≧ 30	0.66	0.08–5.19	0.69			
ALT: < 30 vs. ≧ 30	0.68	0.09–5.35	0.71			

*Staging was based on clinical stage for patients receiving neoadjuvant chemotherapy and on pathological stage for those who did not

**Logistic regression

FN, febrile neutropenia; OR, odds ratio; CI confidence interval; ANC, absolute neutrophil count; ALC, absolute lymphocyte count; AST, aspartate aminotransferase; ALT, alanine aminotransferase

## Supplementary Information

Below is the link to the electronic supplementary material.

Supplementary Material 1

Supplementary Material 2

## Data Availability

The datasets used and/or analyzed during the current study are available from the corresponding author on reasonable request.
