# Supplementary material for: Risk of febrile neutropenia in breast cancer chemotherapy despite the prophylactic use of pegfilgrastim: a retrospective analysis
Source: Breast Cancer. 2026 Aug 14;33(6):1305–11. doi: 10.1007/s12282-026-01907-7 (PMC13695627; doi:10.1007/s12282-026-01907-7)
Supplement: Supplementary file 1 [file 12282_2026_1907_MOESM1_ESM.docx]

| Supplementary Table 1. Patient Characteristics and FN Outcomes Stratified by Prophylaxis | | | |
| --- | --- | --- | --- |
| Variables | Primary  prophylaxis (N=272)  Cases (%) | Secondary prophylaxis (N=106)  Cases (%) | p-value |
| Mean age, years (range) | 53 (23–80) | 52 (27–82) | 0.63 |
| Age |  |  |  |
| < 65 years | 224 (82.4) | 85 (80.2) |  |
| ≥ 65 years | 48 (17.6) | 21 (19.8) | 0.63 |
| Stage* |  |  |  |
| Ⅰ | 47 (17.3) | 20 (18.9) |  |
| Ⅱ | 134 (49.3) | 58 (54.7) |  |
| Ⅲ | 67 (24.6) | 23 (21.7) |  |
| Locoregional recurrence | 24 (8.8) | 5 (4.7) | 0.47 |
| Subtype |  |  |  |
| Luminal | 127 (46.7) | 62 (58.5) |  |
| HER2 positive | 70 (25.7) | 9 (8.5) |  |
| TNBC | 75 (27.6) | 35 (33.0) | <0.01 |
|  |  |  |  |
| Baseline laboratory data median (range) |  |  |  |
| White blood cell count (/μL) | 5500 (3200–10800) | 5900 (2500–10700) | 0.08 |
| Neutrophil count (/μL) | 3380.8 (1320.2–7811.0) | 3596.8 (1014.0–9110.0) | 0.36 |
| Lymphocyte count (/μL) | 1599.6 (483.6–4380.3) | 1664.2 (540.0–3009.0) | 0.07 |
| Hemoglobin (g/dL) | 13.2 (8.3–16.9) | 13.1 (6.8–15.9) | 0.59 |
| Platelet (×10⁴/μL) | 246.5 (61.0–635.0) | 256.5 (144.0–537.0) | 0.23 |
| AST (U/L) |  |  |  |
| < 30 | 249 (91.5) | 90 (84.9) |  |
| ≥ 30 | 23 (8.5) | 16 (15.1) | 0.06 |
| ALT (U/L) |  |  |  |
| < 30 | 252 (92.6) | 88 (83.0) |  |
| ≥ 30 | 20 (7.4) | 18 (17.0) | 0.01 |
| Cardiovascular disease |  |  |  |
| No | 269 (98.9) | 103 (97.2) |  |
| Yes | 3 (1.1) | 3 (2.8) | 0.36 |
| Renal disease |  |  |  |
| No | 268 (98.5) | 106 (100.0) |  |
| Yes | 4 (1.5) | 0 (0.0) | 0.58 |
| Liver disease |  |  |  |
| No | 269 (98.9) | 105 (99.1) |  |
| Yes | 3 (1.1) | 1 (0.9) | 1.00 |
| Diabetes Mellitus |  |  |  |
| No | 259 (95.2) | 103 (97.2) |  |
| Yes | 13 (4.8) | 3 (2.8) | 0.57 |
| Purpose of chemotherapy |  |  |  |
| Neo adjuvant | 171 (62.9) | 69 (65.1) |  |
| Adjuvant | 77 (28.3) | 32 (30.2) |  |
| Locoregional recurrence | 24 (8.8) | 5 (4.7) | 0.79 |
| Regimen |  |  |  |
| Dose dense | 133 (48.9) | 78 (73.6) | <0.01 |
| Tri weekly | 60 (22.1) | 6 (5.7) | <0.01 |
| Chemotherapy with HER2-targeted therapy | 68 (25.0) | 9 (8.5) | <0.01 |
| Chemotherapy with Immune checkpoint inhibitor | 9 (3.3) | 13 (12.3) | <0.01 |
| Event |  |  |  |
| FN with pegfilgrastim | 4 (1.5) | 4 (3.8) | 0.23 |
| Grade 3-4 Neutropenia | 9 (3.3) | 5 (4.7) | 0.55 |

*Staging was based on clinical stage for patients receiving neoadjuvant chemotherapy and on pathological stage for those who did not.

Aberrations: HER2, human epidermal growth factor receptor 2; TNBC, triple negative breast cancer; AST, Aspartate Aminotransferase; ALT, Alanine Aminotransferase
