# Supplementary material for: Risk of febrile neutropenia in breast cancer chemotherapy despite the prophylactic use of pegfilgrastim: a retrospective analysis
Source: Breast Cancer. 2026 Aug 14;33(6):1305–11. doi: 10.1007/s12282-026-01907-7 (PMC13695627; doi:10.1007/s12282-026-01907-7)
Supplement: Supplementary file 2 [file 12282_2026_1907_MOESM2_ESM.docx]

| Supplementary Table 2. Univariate and multivariate analyses of clinical factors associated with FN + severe neutropenia with pegfilgrastim, excluding locoregional recurrence cases (N = 350) | | | | | | | |  |
| --- | --- | --- | --- | --- | --- | --- | --- | --- |
|  |  | Univariate |  |  |  | Multivariate |  | |
|  | OR | 95% CI | *P* value** |  | OR | 95% CI | *P* value** | |
| Age: continuous (per 1 year) | 1.05 | 1.00–1.10 | 0.04 |  | 1.04 | 0.99-1.09 | 0.15 | |
| Stage*: 3- (ref) vs 1-2 |  |  |  |  |  |  |  | |
| Chemotherapy History: No(ref) vs Yes |  |  |  |  |  |  |  | |
| Adjuvant: Adjuvant(ref) vs NAC | 0.71 | 0.23–2.24 | 0.56 |  |  |  |  | |
| Regimen: Dose dense(ref) |  |  |  |  |  |  |  | |
| Tri-weekly | 0.84 | 0.09–7.63 | 0.87 |  |  |  |  | |
| Anti-HER2 | 3.73 | 0.97–14.31 | 0.05 |  |  |  |  | |
| ICI | 5.11 | 0.88–29.72 | 0.07 |  |  |  |  | |
| ANC: continuous (per 1/μL) | 1.00 | 1.00–1.00 | 0.07 |  | 1.00 | 1.00–1.00 | 0.36 | |
| ALC: continuous (per 1/μL) | 1.00 | 1.00–1.00 | 0.59 |  |  |  |  | |
| Hemoglobin: continuous (per 1 g/dL) | 1.02 | 0.65–1.61 | 0.93 |  |  |  |  | |
| Platelet: continuous (per 1 ×10⁴/μL) | 0.98 | 0.97–0.99 | 0.01 |  | 0.98 | 0.97–1.00 | 0.01 | |
| AST: < 30 vs ≧ 30 | 0.72 | 0.09–5.70 | 0.75 |  |  |  |  | |
| ALT: < 30 vs ≧ 30 | 0.72 | 0.09–5.70 | 0.75 |  |  |  |  | |

*Staging was based on clinical stage for patients receiving neoadjuvant chemotherapy and on pathological stage for those who did not.
Abbreviations: FN, febrile neutropenia; OR, odds ratio; CI confidence interval; ANC, absolute neutrophil count; ALC, absolute lymphocyte count; AST, aspartate aminotransferase; ALT, alanine aminotransferase; **Logistic regression
NE, not estimable.
